# First record of the family Gonibregmatidae Cook, 1896 from China (Chilopoda, Geophilomorpha)

**DOI:** 10.3897/BDJ.14.e217565

**Published:** 2026-10-30

**Authors:** Chenlu Yang, Chunxue You, Chao Jiang

**Affiliations:** 1 Tianjin Key Laboratory of Agricultural Animal Breeding and Healthy Husbandry, College of Animal Science and Veterinary Medicine, Tianjin Agricultural University, Tianjin 300392, Tianjin, China Tianjin Key Laboratory of Agricultural Animal Breeding and Healthy Husbandry, College of Animal Science and Veterinary Medicine, Tianjin Agricultural University, Tianjin 300392 Tianjin China; 2 State Key Laboratory for Quality Ensurance and Sustainable Use of Dao-di Herbs, National Resource Center for Chinese Materia Medica, China Academy of Chinese Medical Sciences, Beijing 100700, China State Key Laboratory for Quality Ensurance and Sustainable Use of Dao-di Herbs, National Resource Center for Chinese Materia Medica, China Academy of Chinese Medical Sciences Beijing 100700 China

**Keywords:** China, distribution, key, morphology

## Abstract

**Background:**

Gonibregmatidae is a poorly known family of soil centipedes in Geophilomorpha. The genus *Vinaphilus* is known from Vietnam, but its diversity and distribution remain insufficiently documented. Here, we report the first record of *Vinaphilus unicus* from China, providing new information on the distribution of Gonibregmatidae and the species.

**New information:**

The family Gonibregmatidae Cook, 1896 is newly recorded from China, based on specimens of *Vinaphilus unicus* Tran, Tran & Bonato, 2019 collected from Hainan Province. The male of this species is described for the first time, with particular attention to the postpedal structures. Detailed descriptions, illustrations and distribution data are provided.

## Introduction

Gonibregmatidae Cook, 1896 is a species-poor and poorly known family of soil centipedes within Geophilomorpha (Cook 1896; Bonato et al. 2011). The family includes some of the largest and most elongate geophilomorphs, with adults of several species exceeding 10 cm in length (JGE 1981; Bonato et al. 2011; Tran et al. 2019). It also includes *Gonibregmatus plurimipes* Chamberlin, 1920, a species endemic to Fiji which possesses 191 pairs of legs, the highest number recorded in Chilopoda (Minelli and Bortoletto 1988; Arthur et al. 2002; Bonato et al. 2010; Bonato et al. 2011; Tran et al. 2019). Under the relatively narrow circumscription adopted by Tran et al. (2019) and including the two species subsequently described by Tsukamoto et al. (2021), Gonibregmatidae comprises 10 genera and 19 described species. Most species are known from the Indo-Australasian Region, with records extending from the Indian Peninsula through the Indochinese Peninsula and the Malay Archipelago to New Guinea and the Fijian Islands (Bonato et al. 2011; Tran et al. 2019; Tsukamoto et al. 2021). Prior to the present study, no species of Gonibregmatidae had been recorded from China.

The genus *Vinaphilus* Tran, Tran & Bonato, 2019 was originally established for *V. unicus* Tran, Tran & Bonato, 2019 from northern Vietnam (Tran et al. 2019). The genus is characterised by a unique tripartite arrangement of ventral pore fields, comprising two anterior sublateral fields and one medial posterior field on most metasternites (Tran et al. 2019). Two additional species, *V. chuyangsinensis* Tsukamoto, Nguyen & Eguchi, 2021 and *V. dentatus* Tsukamoto, Nguyen & Eguchi, 2021, were subsequently described from the Central Highlands of Vietnam (Tsukamoto et al. 2021). The original description of *V. unicus* was based exclusively on two females from a single locality in Vinh Phuc Province; consequently, its male morphology and distribution beyond the type locality remained unknown (Tran et al. 2019). Based on male specimens collected from Hainan Island in February 2021, we herein report the first records of Gonibregmatidae and *Vinaphilus* from China, extend the known distribution of *V. unicus* beyond Vietnam and describe the male morphology of the species for the first time.

## Materials and methods

Specimens were collected by hand using tweezers from under decaying wood in Jianfengling National Forest Park, Hainan Province, China, on 24 February 2024. The specimens are preserved in 75% ethanol and deposited at the National Resource Center for Chinese Materia Medica, China Academy of Chinese Medical Sciences, Beijing, China (**CMMI**).

Specimens were dissected and the cephalic capsules, mandibles and maxillary complexes were mounted on temporary slides with 75% ethanol. To examine the coxal pores, samples were cleared in 75% lactic acid for approximately 5–7 hours until the internal structures became visible under a light microscope. Morphological examination and imaging were carried out using a Leica M205 FCA stereomicroscope and an Olympus BX51 microscope. Photographs were converted into hand-drawn illustrations with SKETCHBOOK 6.2.3.

Morphological terminology for external anatomy follows that of Bonato et al. (2010).

## Taxon treatments

### Vinaphilus unicus

Tran, Tran & Bonato, 2019

51654914-7628-52EA-AB6A-7E0B949FAD23

#### Materials

- **Type status:** Other material. **Occurrence:** catalogNumber: 20240224006D; recordedBy: Chao Jiang; individualCount: 1; sex: male; lifeStage: adult; occurrenceID: 401BAAD2-3997-5E0A-8604-6343A05E6A56; **Taxon:** scientificName: Vinaphilus unicus; **Location:** country: China; stateProvince: Hainan; locality: Jianfengling National Forest Park; verbatimElevation: 1150 m; verbatimCoordinates: 18.7148°N, 108.8761°E; **Event:** eventDate: 24.ii.2024; **Record Level:** language: en; institutionCode: CMMI
- **Type status:** Other material. **Occurrence:** catalogNumber: 20240224007D; recordedBy: Chao Jiang; individualCount: 1; sex: male; lifeStage: adult; occurrenceID: CE7CE95D-CA6B-5151-97A7-7F8E03421CEF; **Taxon:** scientificName: Vinaphilus unicus; **Location:** country: China; stateProvince: Hainan; locality: Jianfengling National Forest Park; verbatimElevation: 1150 m; verbatimCoordinates: 18.7148°N, 108.8761°E; **Event:** eventDate: 24.ii.2024; **Record Level:** language: en; institutionCode: CMMI

#### Description

**Figure 1.**
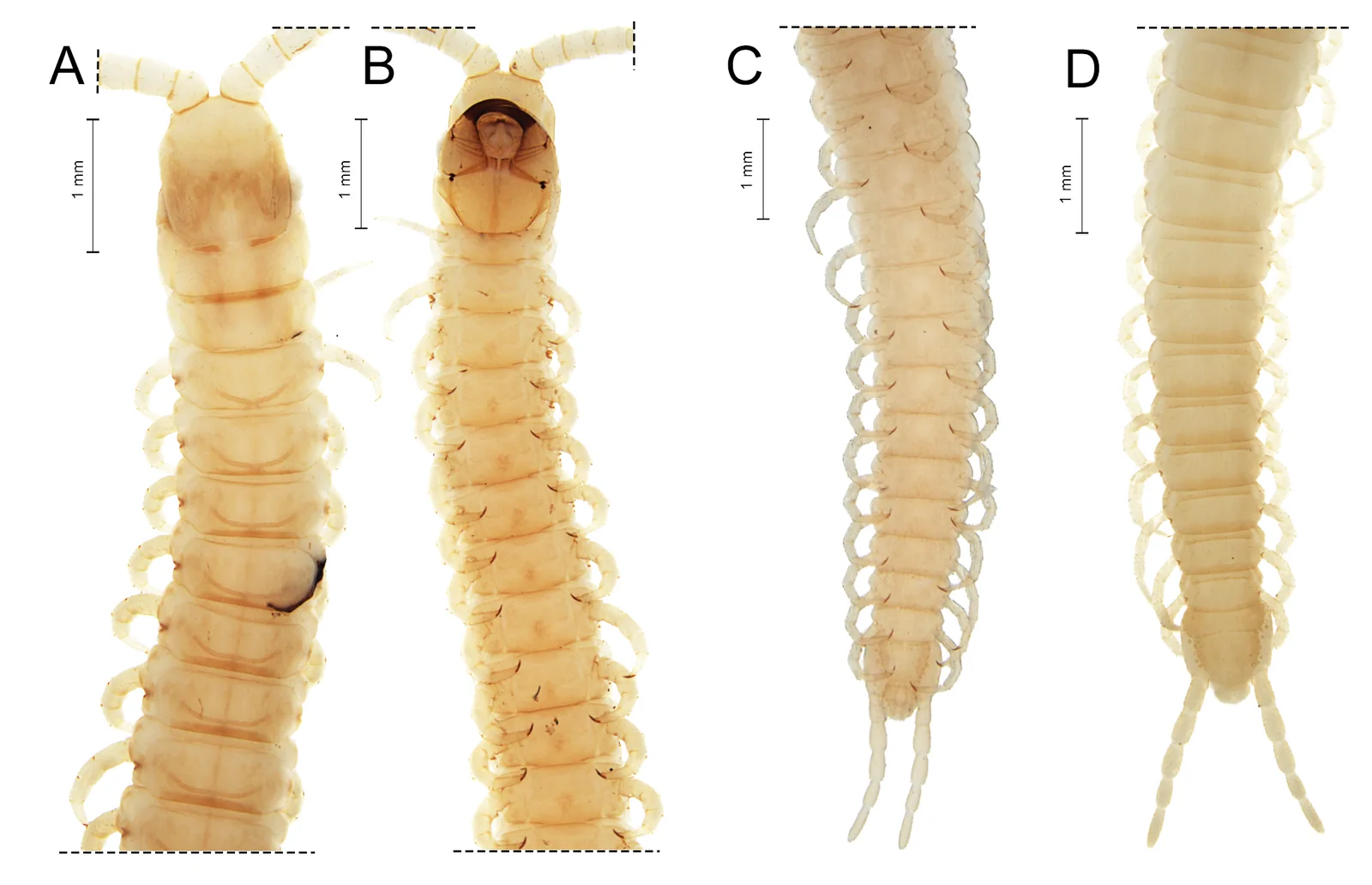
*Vinaphilus unicus* Tran, Tran & Bonato, 2019. Spm. 20240224007D. **A** anterior part of the body, dorsal view; **B** anterior part of the body, ventral view; **C** posterior part of the body, dorsal view; **D** posterior part of the body, ventral view. Scale bars: 1 mm.

***General features***. (Fig. 1A–D) Body length of males 39–51 mm, with 85–87 leg-bearing segments. Specimen 20240224006D measures 39 mm and has 87 leg-bearing segments; specimen 20240224007D measures 51 mm and has 85 leg-bearing segments. Body nearly the same width anteriorly and posteriorly, becoming gradually narrower only at last 5–7 segments. Colour (in 75% ethanol) yellowish; forcipules darker.

***Cephalic capsule*** (Fig. 3A) subrectangular, length approximately 1.2 times width; anterior margin slightly convergent and rounded; lateral margins straight, nearly parallel to each other; posterior margin nearly straight, lacking distinct concavities or projections; all four corners smoothly rounded, without sharp angles. Longer setae scattered on anterolateral portions of cephalic plate.

**Figure 2.**
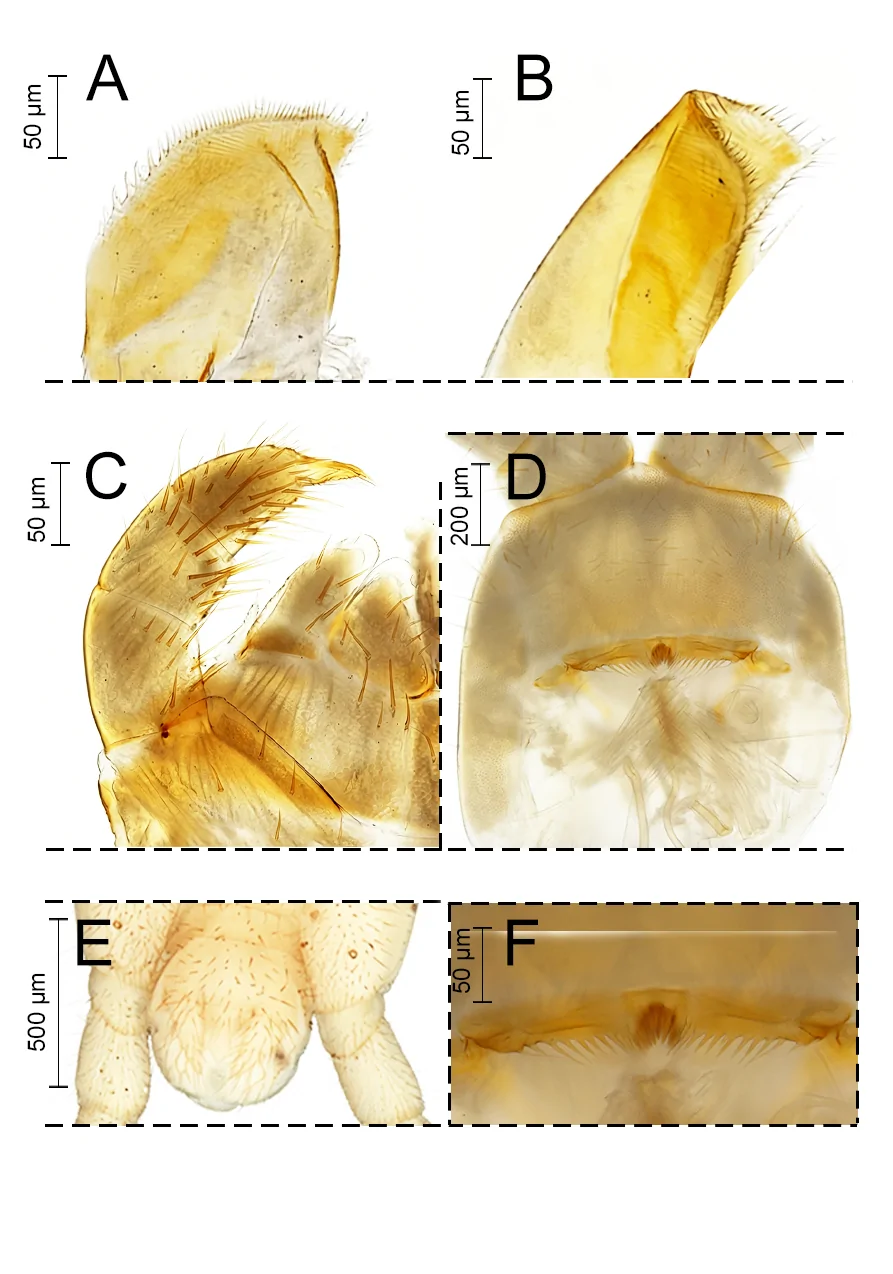
*Vinaphilus unicus* Tran, Tran & Bonato, 2019. Spm. 20240224007D. **A** Left mandible, dorsal view; **B** Left mandible, ventral view; **C** Maxillary complex, ventral view (partial detail); **D** Anterior part of cephalic capsule, ventral view (forcipules, maxillae and mandibles removed); **E** Posterior end of body in adult male, ventral view, gonopods detail; **F** Labrum, ventral view.

**Figure 3.**
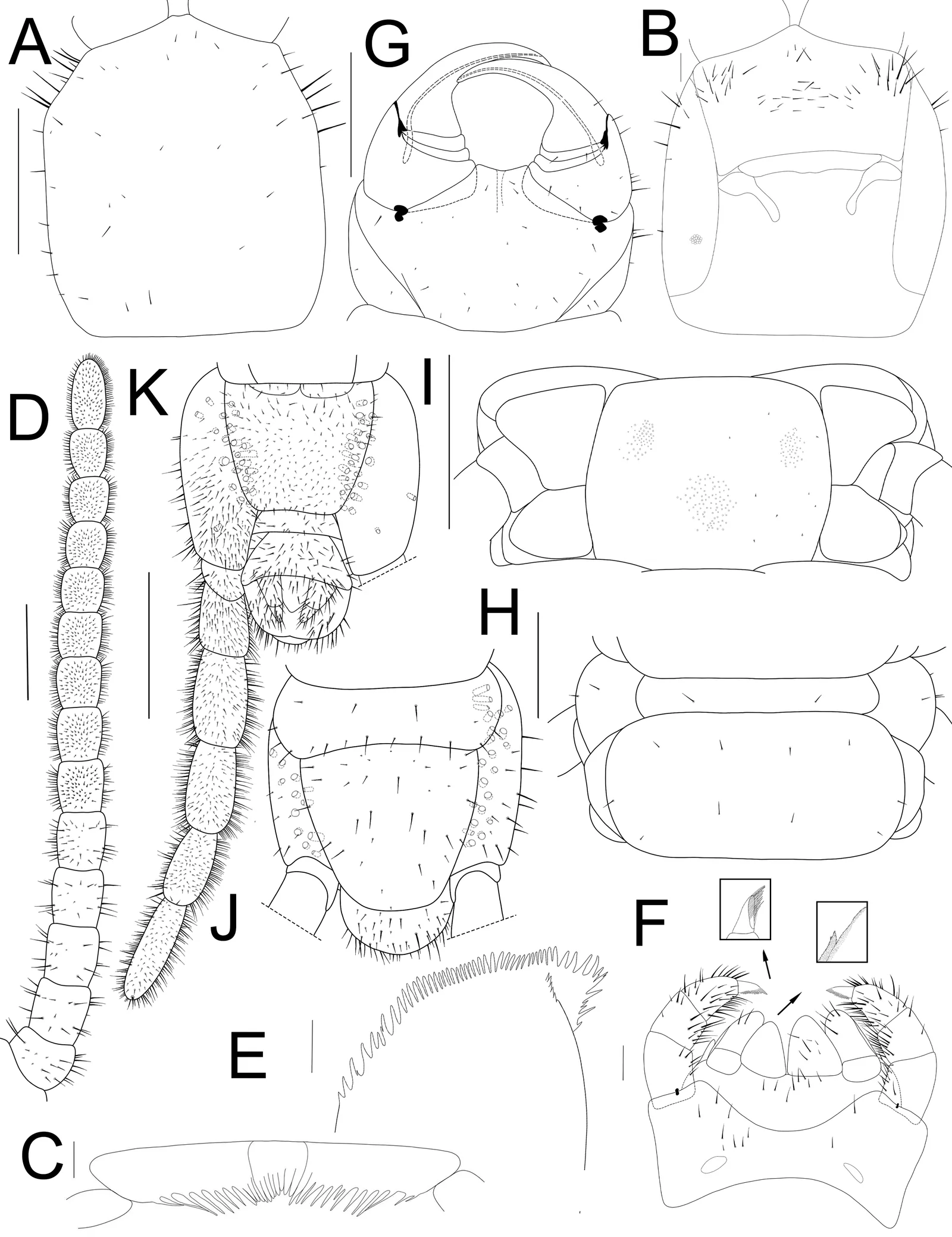
*Vinaphilus unicus* Tran, Tran & Bonato, 2019. Specimen: 20240224007D. **A** Cephalic plate, dorsal view; **B** Anterior part of cephalic capsule, ventral view; **C** Labrum, ventral view; **D** Left antenna, ventral view; **E** Mandible; **F** Maxillary complex, ventral view; **G** Forcipular segment; **H** Tergite of leg-bearing segment, dorsal view; **I** Sternum of the middle trunk segments, ventral view (setae on right part omitted); **J** Posterior end of body in adult male, dorsal view; **K** Posterior end of body in adult male, ventral view. Scale bars: 500 μm (**A, D, G–K**); 100 μm (**B, F**); 50 μm (**C**); 20 μm (**E**).

***Clypeus and labrum*** (Fig. 2D and F; Fig. 3B and C) Clypeus ca. 1.8 times as wide as long, with lateral margins; no obvious clypeal areas; at least 57 setae, all in the anterior half of the clypeus, most of them in a subtriangular intermediate broad area, ca. 12 setae in each of 2 anterolateral smaller areas and a few other apparently broken setae close to the mid-point of the anterior margin of the clypeus. Pleurites uniformly areolate. Labral margin slightly concave, with 8 median denticles and 11–13 lateral projections on each side; the denticles darker, shorter and more closely spaced than the lateral projections; the denticles are darker, shorter and closer to each other than the lateral projections.

***Antennae*** (Fig. 3D) composed of 14 articles. Basal articles 1–5 with sparse long setae. From ca. 6 article onwards, setae much shorter and denser. Intermediate articles about as long as wide. Ultimate article distinctly elongate, ca. 1.5 times as long as wide, ca. 1.3 times as long as the penultimate article.

***Mandible*** (Fig. 2A and B; Fig. 3E) A single pectinate lamella, with 20–30 elongate teeth. Each tooth about 4.5 times as long as wide.

***First maxillae*** (Fig. 2C; Fig. 3F) Coxosternite entire, with setae close to the anterior margin; a pair of lappets, covered with scales, a pair of lappets, each covered with scales and ca. 2 times as long as the telopodital lappet. Coxal projection subtriangular, longer than wide, with setae on the ventral side. Telopodite longer than wide, of 2 articles, with setae on the ventral side; a lappet emerging from the basal article and covered with scales, ca. 0.9× as long as the basal article.

***Second maxillae*** (Fig. 2C; Fig. 3F) Coxosternite entire, with anterior margin deeply angulated, metameric pores on the central part of each half and about 7 setae near each of the posterior corners. Telopodite of 3 articles, slightly narrowing towards the tip; a number of setae on each article, most of them on the meso-ventral side; pretarsus slightly bent, narrowing and slightly spoon-shaped at the tip, bearing two marginal rows of fine filaments; associated sensilla could not be reliably resolved in the available preparations.

***Forcipular segment*** (Fig. 3G) Tergite subtrapezoidal, ca. 2.8 times as wide as long, with lateral margins strongly converging anteriorly, approximately as wide as the cephalic plate and similar in length and width to the following tergite. The exposed part of the coxosternite is ca. 1.3 times as wide as long; its anterior margin with a shallow medial concavity and without denticles; complete coxopleural sutures, entirely ventral, sinuous and diverging anteriorly; chitin-lines incomplete, only visible in the posterior part of the coxosternite. Trochanteroprefemur ca. 1.4 times as wide as long. No denticles along the forcipule. Tarsungulum with both external and internal margins uniformly curved, except for a moderate mesal basal bulge; internal margin finely serrate. Poison calyx present.

***Leg-bearing segment*** (Fig. 3H and I) Metatergite 1 about as wide as the subsequent one, without pretergite. No paratergites. Legs of the first pair slightly smaller than the following ones; claws simple, uniformly bent, with 2 accessory spines. Ventral glandular pores densely grouped into 3 separate fields on each metasternite, from the second to the prepenultimate leg-bearing segments: 2 paired fields in the anterior part of the metasternite, near the lateral margins. These fields are subcircular and closer to the anterior corners in the most anterior segments, whereas they are slightly elongated longitudinally and closer to mid-length in the remaining trunk; 1 subcircular medial field approximately in the centre of the metasternite in the anterior part of the body, gradually becoming closer to the posterior margin in the most posterior segments. Only the medial field present on the first and the penultimate leg-bearing segment.

***Ultimate leg-bearing segment*** (Fig. 3J and K) Pleuropretergite entire, without sulci. Metatergite subtrapezoidal, wider than long, with lateral margins convex and converging posteriorly. Approximately 22 coxal pores observed on the ventral side of each coxopleuron, most clustered and concealed by the metasternite. Additional pores present dorsally, partly concealed by the metatergite, with some remaining exposed. Metasternite subtrapezoidal, wider than long, anteriorly ca. 1.8 times as wide as posteriorly, with lateral margins slightly convex and converging posteriorly; covered with setae of varying lengths, more densely distributed near the posterior margin. Ultimate telopodite composed of 6 articles, including 2 tarsal articles, ca. 13 times as long as wide and ca. 2 times as long and ca. 2 times as wide as the penultimate telopodite. Tarsus 2 ca. 1.2 times as long as wide and ca. 1.7 times as long as tarsus 1. Setae uniformly dense on most of the ventral and dorsal surfaces of the telopodite, less than 70 μm long. Pretarsus absent, replaced by a small terminal spine.

***Postpedal segments*** (Fig. 2E; Fig. 3J and K) Male gonopods paired, bi-articulate, situated on either side of the penis and extending posteriorly. Basal article broad; distal article narrower, with a rounded apex. Both articles densely setose, with long setae extending beyond their margins. Penis conical, situated medially between the gonopods. Anal organs not observed.

#### Diagnosis

Body length 39–51 mm in males and 65–90 mm in females; number of leg-bearing segments 85–87 in males and 109 in both known females. Head and forcipular segment relatively short; cephalic plate without a transverse suture. Forcipular coxosternite with incomplete chitin-lines. Ventral pore-fields comprising two anterior sublateral fields and one posterior median field on metasternites of leg-bearing segments 2 to the prepenultimate; only the median field present on the first and penultimate leg-bearing segments. Metasternite of the ultimate leg-bearing segment subtrapezoidal and wider than long. Most coxal pores concealed by the metasternite of the ultimate leg-bearing segment. Telopodites of the ultimate legs each bearing a small terminal spine (modified from Tran et al. (2019)).

#### Distribution

**Figure 4.**
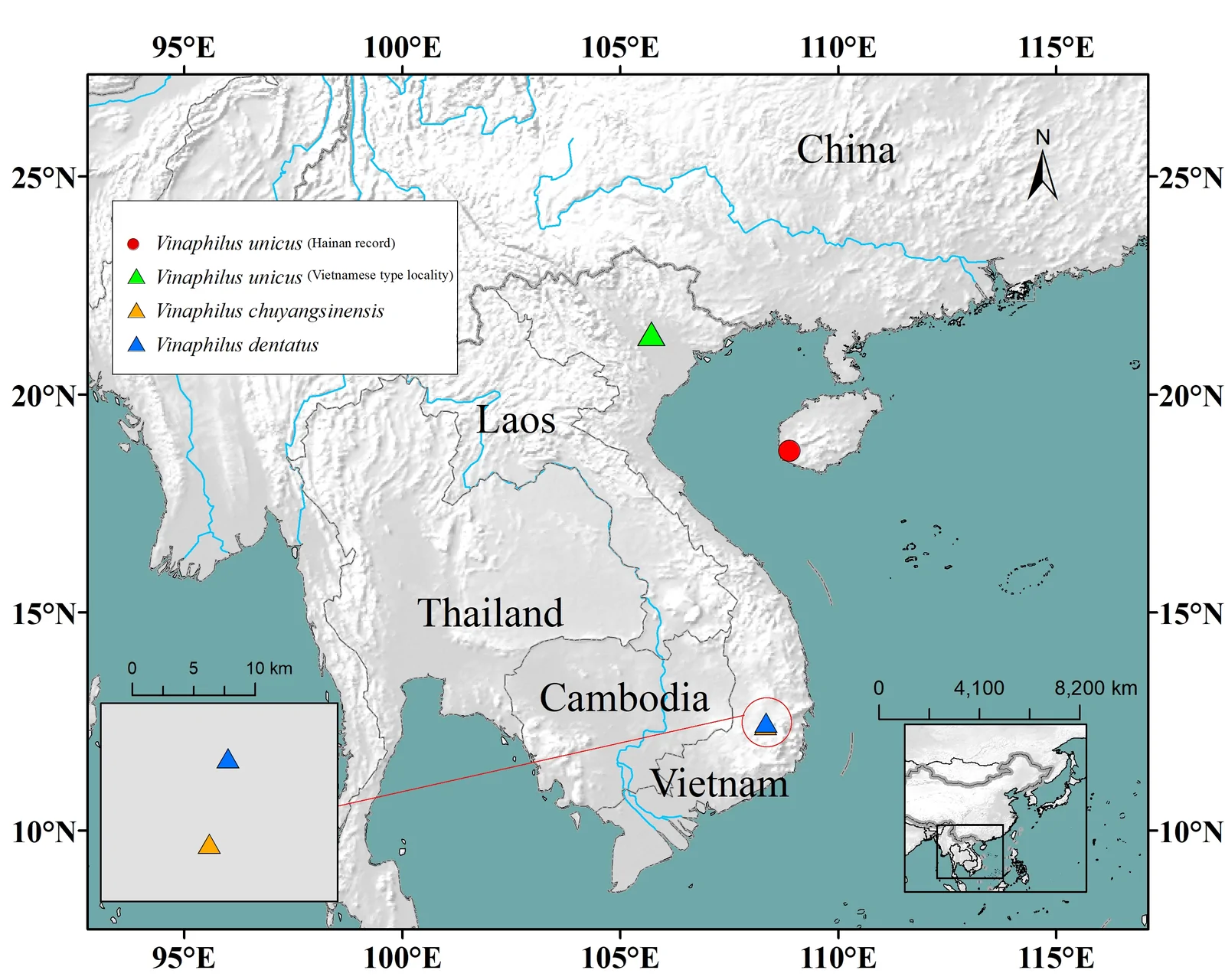
Localities of *Vinaphilus* species. Circles represent specimens examined in this study (*V. unicus* from Hainan). Triangles represent type localities: one in northern Vietnam for *V. unicus* and two in the Central Highlands of Vietnam for *V. chuyangsinensis* and *V. dentatus*. The lower-left inset shows the two closely spaced localities in central Vietnam. The red circle and connecting line indicate their approximate location on the main map.

Vinh Phuc Province, northern Vietnam; China, Hainan Province (Fig. 4).

## Identification Keys

### Key to the three species of *Vinaphilus* described in Tran et al. (2019) and Tsukamoto et al. (2021)

**Table:** 

1	Anterior margin of forcipular coxosternite with a pair of denticles	*V. dentatus*
–	Anterior margin of forcipular coxosternite without denticles	2
2	Chitin-lines of forcipular coxosternite complete	*V. chuyangsinensis*
–	Chitin-lines of forcipular coxosternite incomplete	*V. unicus*

## Discussion

The Hainan specimens examined in this study are confidently assigned to *Vinaphilus* Tran, Tran & Bonato, 2019, as they share the key diagnostic character of the ventral pore-fields, which is unique to this genus. The specimens agree with *V. unicus* in the combination of the characteristic arrangement of the ventral pore fields, incomplete chitin-lines on the forcipular coxosternite and absence of denticles along its anterior margin (Tran et al. 2019; Tsukamoto et al. 2021). This combination distinguishes *V. unicus* from its two congeners. See Table 1.

Nevertheless, the Hainan population differs from the type population (northern Vietnam) of *V. unicus* in several quantitative characters. The body length of the Hainan specimens ranges from 39 to 51 mm, distinctly smaller than the holotype (65 mm) and paratype (90 mm). The number of leg-bearing segments is 85–87 in the Hainan population, compared with invariable 109 in the Vietnamese population. Furthermore, the setae on the metasternite of the ultimate leg-bearing segment are distinctly denser near the posterior margin in the Hainan specimens, whereas they were described as almost absent there in the type material. Several other quantitative differences also exist: each coxopleuron bears ca. 22 coxal pores in the Hainan specimens, compared with ca. 30 in the holotype and ca. 40 in the paratype described by Tran et al. (2019); the cephalic plate is ca. 1.2 times as long as wide (vs. roughly as long as wide in the original), the clypeus is ca. 1.8 times as wide as long (vs. ca. 2.7 times) and the forcipular coxosternite is ca. 1.3 times as wide as long (vs. ca. 1.7 times). As the type series consists exclusively of females, whereas all Hainan specimens are males and females in geophilomorph centipedes generally possess more leg-bearing segments than males (Brena et al. 2013), the observed differences may reflect sexual dimorphism, geographic variation or a combination of both. However, only two males from Hainan and two females from northern Vietnam are available and sex is completely confounded with geographical origin. This small sample prevents us from determining whether the observed differences represent consistent sexual dimorphism, geographical differentiation or other intraspecific variation. Examination of additional specimens of both sexes from the same localities, ideally supplemented by molecular evidence, will be necessary to clarify the extent and sources of variation within *V. unicus*.

The Hainan specimens were collected beneath decaying wood at approximately 1,150 m asl., whereas the Vietnamese types were found in secondary forest at approximately 150 m (Tran et al. 2019). Forest type in Hainan and microhabitat in Vietnam remain undocumented, limiting ecological comparison.

The present finding represents the first records of the genus *Vinaphilus* as well as the family Gonibregmatidae from China and extends the known distribution of *V. unicus* from northern Vietnam to Hainan Island. Together with the occurrence of two additional congeners in Vietnam (Tsukamoto et al. 2021), this record indicates that the diversity and distribution of *Vinaphilus* in Southeast Asia and southern China remain incompletely documented. The apparent geographical gaps may partly reflect insufficient sampling of soil-dwelling arthropods rather than true distributional discontinuities. However, the limited records do not permit conclusions about historical dispersal, geographical isolation or population differentiation. Further targeted sampling in southern China, including Hainan and intervening areas of Southeast Asia would help clarify the distribution of the genus.

## Supplementary Material

XML Treatment for Vinaphilus unicus

## Figures and Tables

**Table 1.** Comparison of selected morphological characters of three species of *Vinaphilus*, with separate data for the Hainan males and Vietnamese females of *V. unicus* (Tran et al. 2019; Tsukamoto et al. 2021).

Character	*V. unicus*: Hainan males	*V. unicus*: Vietnamese females	*V. chuyangsinensis*	*V. dentatus*
Material reported	2 adult males	2 adult females	2 adult females and 1 immature specimen	1 adult male
Adult body length (mm)	39–51	65–90	77–80	35
Leg-bearing segments	85–87	109	97–105	87
Cephalic plate length/width	Ca. 1.2	Ca. 1.0	Ca. 1.0	Ca. 1.2
Forcipular chitin-lines	Incomplete	Incomplete	Complete	Incomplete
Denticles on anterior margin of forcipular coxosternite	Absent	Absent	Absent	One pair
Filaments on second maxillary pretarsus	Two marginal rows	Two marginal rows	7 on dorsal margin and 7 on ventral margin	17 along margin
Sensilla on second maxillary pretarsus	Not reliably resolved	Present	4 pore-like sensilla per pretarsus	Not visible
Internal margin of tarsungulum	Finely serrate	Finely serrate	Serrate	Weakly serrate
General arrangement of sternal pore-fields	Two anterior sublateral fields and one posterior median field	Two anterior sublateral fields and one posterior median field	Two anterior sublateral fields and one posterior median field	Two anterior sublateral fields and one posterior median field
Shape of anterior paired pore-fields	Subcircular anteriorly, becoming longitudinally elongated posteriorly	Subcircular anteriorly, becoming longitudinally elongated posteriorly	Subcircular on segments 2–5, longitudinally elongated thereafter	Subcircular on segment 2, longitudinally elongated thereafter
Coxal pore count per coxopleuron	Ca. 22 observed ventrally; additional dorsal pores present	Ca. 30 in holotype; ca. 40 in paratype	Ca. 190–230	Ca. 26–27
Distribution of coxal pores	Dorsal and ventral; dorsal pores partly concealed by metatergite	Dorsal and ventral; dorsal pores partly concealed by metatergite	Predominantly mesodorsal and mesoventral	Predominantly mesodorsal and mesoventral
Ultimate pretarsus	Absent; small terminal spine present	Absent; small terminal spine present	Absent	Absent
Source	Present study	Tran et al. (2019)	Tsukamoto et al. (2021)	Tsukamoto et al. (2021)
